# Anthocyanins^☆^

**DOI:** 10.1016/j.advnut.2026.100692

**Published:** 2026-06-30

**Authors:** Taylor C Wallace, M Monica Guisti

**Affiliations:** 1 Think Healthy Group, Inc., Washington, DC, United States; 2 School of Medicine and Health Sciences, George Washington University, Washington, DC, United States; 3 Friedman School of Nutrition Science and Policy, Tufts University, Boston, MA, United States; 4 Department of Food Science & Technology, The Ohio State University, Columbus, OH, United States

## Anthocyanins

**FIGURE 1:**
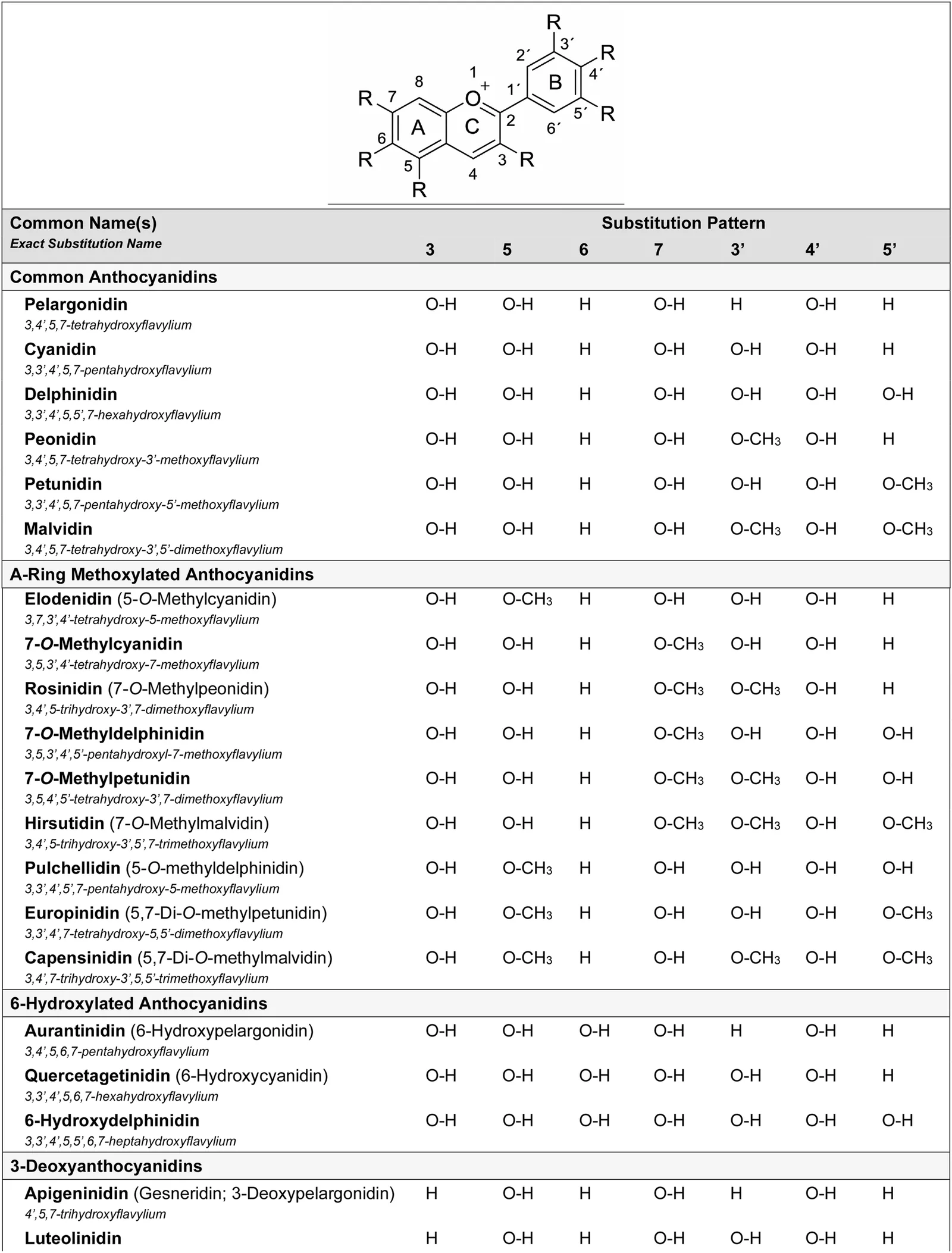

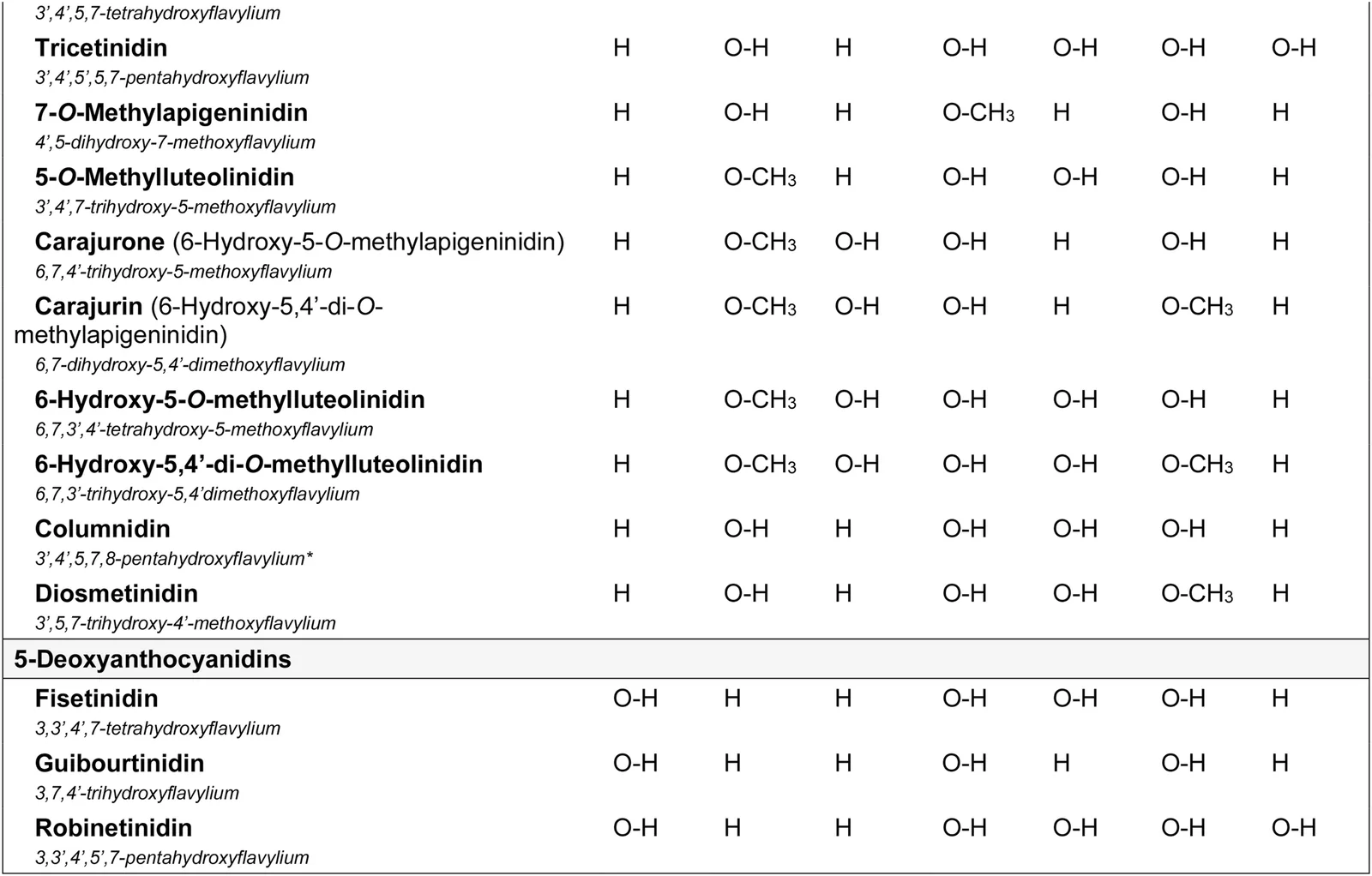
Chemical structures of the 32 naturally occurring anthocyanidins with a (2-phenylbenzopyrylium) C6–C3–C6 carbon skeleton.

Anthocyanins (from the Greek *anthos*, meaning “flower,” and *kyáneos*, meaning “blue”) are polyphenolic compounds belonging to the flavonoid subclass of specialized secondary plant metabolites. They are synthesized by plants for a variety of reasons, often in response to various environmental stressors, and are responsible for many of the vivid red, purple, and blue hues (maximum absorption in the visible range is usually between 465 and 550 nm) observed in many fruits, vegetables, flowers, leaves, and other plant tissues. At least 900 structurally distinct anthocyanins are currently known to exist in nature [1,2]. These anthocyanins are derivatives of 32 known aglycons (commonly known as “anthocyanidins”), although 6 common anthocyanidins—cyanidin, delphinidin, pelargonidin, peonidin, petunidin, and malvidin—serve as the backbone for ∼90% of all anthocyanins identified to date (Figure 1) [1,3]. Anthocyanidins contain a C_15_ (C_6_–C_3_–C_6_) carbon skeleton, often referred to by chemists as a 2-phenylbenzopyrylium or flavylium cation, each uniquely hydroxylated and/or methoxylated in various positions, most commonly at C_3_, C_5_, C_6_, C_7_, and C_3_′, C_4_′, and C_5_′. Free (liberated) anthocyanidins are chemically unstable and therefore relatively uncommon in nature. Consequently, the vast majority of anthocyanins (∼90%)—with the notable exception of 3-deoxyanthocyanins—occur as *O*-glycosides bearing ≥1 glucosyl moieties, most commonly attached at the C_3_ hydroxyl group but also potentially at C_5_, C_7_, C_3_′, C_4_′, and/or C_5_′ [1,3]. Further contributing to their structural diversity and enhancing their chemical stability, ∼70% of naturally occurring anthocyanins contain aliphatic and/or aromatic acyl groups linked to their sugar moieties [1].

Anthocyanins represent one of the most widely studied groups of dietary bioactive compounds because of their versatility as natural alternatives to synthetic food colorants and their benefits to long-term health maintenance. Consistent human evidence supports that higher intakes of anthocyanins may help prevent or ameliorate several age-related diseases, including cardiovascular, neurodegenerative, metabolic, skeletal, ocular, and certain forms of cancer, among others.

Their health-protective effects are multifaceted, extending beyond their widely recognized antioxidant and anti-inflammatory properties to their ability to modulate key enzymatic functions and cell signaling pathways involved in vascular and endothelial function, glucose-lipid metabolism, mitochondrial bioenergetics, and gene expression, among others. Near-isogenic food genotypes developed through conventional breeding or metabolic engineering that differ only in their anthocyanin contents have enabled researchers to pinpoint distinct mechanisms of action and synergies attributable specifically to anthocyanins [4]. Although intact anthocyanins in the plasma represent ≤1% of the total dietary intake, their breakdown products and gut microbial metabolites (e.g., hydroxybenzoic, hippuric, and hydroxyphenylacetic acids) reveal a much higher bioavailability. For example, the relative bioavailability of cyanidin-3-glucoside, the most prominent anthocyanin found in nature, was shown to be 12.4% ± 1.38% in a ^13^C-tracer study [5]. These breakdown products and microbial metabolites are known to exert distinct modulatory effects on key metabolic and disease-related pathways, providing mechanistic support for the many biomarker-based findings observed among clinical intervention studies and health outcome–based findings among prospective cohort studies.

## Deficiencies

Anthocyanins are not considered classic nutrients but rather dietary bioactive compounds, because their presence in the diet is not required for normal growth, development, physiological function, or survival in humans. Instead, anthocyanins offer value through their potential to support health maintenance, reduce chronic disease risk, and serve as adjuvant dietary therapeutic agents in specific contexts (e.g., cardiovascular disease).

## Diet Recommendations

Currently, no internationally agreed-upon Nutrient Reference Value (NRV) exists for anthocyanins, consistent with other dietary bioactive compounds. China currently recommends a Specific Proposed Level, an NRV akin to the United States Recommended Dietary Allowance, of 50 mg/d of anthocyanins [6]. Similarly, Russian nutritional guidelines recommend a daily intake of 50 to 150 mg to support health and physiological function [7]. Other international and country-specific regulatory and public health authorities, including the WHO, the United States, Canada, Japan, and the European Union, have not yet established evidence-based quantitative intake targets for anthocyanins. The Academy of Nutrition and Dietetics is currently developing a clinical guideline and intake recommendation to address the role of anthocyanins in cardiometabolic health in the adult population.

## Food Sources

Fruits (e.g., apples, berries, cherries, figs, grapes, plums, and pomegranates), vegetables (e.g., eggplant, radish, purple carrot, red cabbage, purple cauliflower, red onion, purple potatoes, and purple corn), legumes (e.g., black soybean, red kidney beans, and black beans), and grains (e.g., black or red rice and purple wheat) are good dietary sources of anthocyanins. Because of their high anthocyanin content and broad consumption, berries such as bilberries, blueberries, blackberries, chokeberries, mulberries, strawberries, and raspberries represent top sources in diets across the globe. Concentrated anthocyanin-rich extracts have continued to gain popularity over the last decade, both as natural alternatives to synthetic colorants, many with expanded functionality in a growing number of packaged food categories, and as ingredients in many dietary supplement products marketed to consumers for a variety of health benefits.

Recent data from the nationally representative United States NHANES (2017–2018 data cycles) indicate blueberries (19%), red wine (includes dessert wine, 11%), flavored milk/other dairy drinks (includes dairy-based smoothies, 9%), grapes (9%), 100% noncitrus fruit juice (not apple, 7%), cranberries (7%), strawberries (6%), nuts and seeds (5%), and blackberries and raspberries (5%) to be top sources of anthocyanins in the diets of adults [8]. In the United Kingdom, recent national retail data show similar patterns, with wine and berries contributing the most anthocyanins to the diet, in addition to other foods such as drupe fruits, 100% fruit juice, berry jams, black olives, leaf vegetables, pulses and beans, and red onions [9]. It is important to note that population-level estimates of exposure rely on the use of food composition databases (Phenol Explorer, USDA Database for the Flavonoid Content of Foods) to derive reference amounts in food. The contents reported among these databases vary on the basis of the methods used to estimate content in food, the completeness of the database, and regional specificity. Anthocyanin content in food is also considerably influenced by seasonality, environmental conditions (temperature, soil quality and acidity, precipitation, altitude and UV exposure, frost, etc.), agricultural practices, and harvest.

## Clinical Uses

In lieu of the forthcoming Academy of Nutrition and Dietetics clinical guideline, there is a general lack of authoritative guidance on the utility of anthocyanins for chronic disease risk reduction. Prospective observational studies suggest a decreased cardiometabolic risk at intake ranges of 20 to 50 mg/d from food, whereas data from clinical trials administering anthocyanins in dietary supplement form suggest therapeutic effects on risk biomarkers at higher doses (80–320 mg/d), depending on the outcome. Recent systematic reviews and clinical trials provide support for cardiovascular and cerebrovascular protection and anticancer (particularly esophageal, gastric, and colon) effects of anthocyanins. A recent systematic review and meta-analysis of prospective cohort studies in healthy adults found that higher anthocyanin intake was associated with 26%, 18%, 11%, 9%, and 8% lower risks of cardiovascular disease (CVD), myocardial infarction (MI), type 2 diabetes, CVD mortality, and hypertension, respectively, whereas the risk of stroke was unaffected. The certainty of the evidence was moderate for the CVD and MI outcomes. Evidence from clinical trials indicated that anthocyanin intakes as low as 50 mg/d improved biomarkers of atherosclerosis and metabolic syndrome in healthy adults, with strong certainty of evidence for the effects of acute anthocyanin intake on flow-mediated dilation (FMD) and moderate certainty of evidence for the effects of chronic intake on FMD [10]. Dietary and supplemental intakes have been demonstrated to improve endothelium-dependent flow-mediated dilation and subsequent vascular function, blood flow to the brain (monitored using functional magnetic resonance imaging), blood lipids (HDL cholesterol, LDL cholesterol, triglycerides, and total cholesterol), glucose-insulin dynamics (fasting blood glucose and hemoglobin A1c), proinflammatory cytokines and chemokines, and biomarkers of oxidative stress, particularly among individuals with elevated concentrations [11]. These data suggest that dietary supplementation and/or encouragement of foods rich in anthocyanins may have promising adjunctive benefits for preventing or treating a number of inflammatory-related chronic conditions, particularly those related to cardiometabolic dysregulation.

## Toxicity

To date, human clinical trials have not identified adverse effects of anthocyanin intake. Anthocyanins exhibit very low acute and subchronic toxicity with no in vivo genotoxic or carcinogenic signal in animal studies, even at high doses. Across multiple species, including rats, mice, guinea pigs, rabbits, and dogs, administration of concentrated anthocyanin-rich extracts at amounts ranging from 20 to 9000 mg/kg body weight/d has not produced toxic effects, with the upper range tested across over 3 generations [3]. A recent 14-d acute toxicity study in Sprague–Dawley rats reported that purified cyanidin exhibited a lethal dose 50% >300 mg/kg body weight/d administered dose [12].

Following a request from the European Commission, the European Food Safety Authority (EFSA) Panel on Food Additives and Nutrient Sources Added to Food undertook a comprehensive re-evaluation of the safety of anthocyanins used as food colorants (E 163). In its 2013 assessment, EFSA concluded that the existing toxicological database was inadequate to establish a numerical adequate daily intake (ADI) for anthocyanins [13]. Most toxicological data originated from grape skin and blackcurrant extracts, the 2 most common anthocyanin-based color additives. EFSA determined these specific extracts were unlikely to pose a safety concern but emphasized that the data were insufficient to generalize across all anthocyanin-containing color additives to support a population-wide safety threshold [13]. This position somewhat contrasts with an earlier (1984) Joint FAO/WHO Expert Committee on Food Additives (JECFA) ADI designation of 0 to 2.5 mg/kg body weight/d for anthocyanins from grape skin extracts for use as color additives only [14]. Both EFSA and JECFA emphasize that ADIs are toxicological safety benchmarks, not nutrient-based recommendations, and therefore should not be interpreted as NRVs or intake targets. Likewise, China and Russia, the only countries to establish intake recommendations for anthocyanins, have also restricted their guidance to the safety of specific additive forms rather than establishing a broader tolerable upper intake level [6,7].

## Recent Research

### Improvements in anthocyanins as functional ingredients

Anthocyanins are highly prone to degradation by various external environmental fluctuations (e.g., temperature, pH, oxygen, light, and enzymes). Enhanced stability of anthocyanins has been achieved through the grafting of new functional groups onto an anthocyanin through chemical reactions that maintain the structure of the precursor compound while improving its chemical stability (e.g., acylation and pyranization). Delivery systems such as nanoencapsulation and microencapsulation have helped to improve the stability, solubility, gastrointestinal permeability, and overall bioavailability of these now commercially available ingredients by utilizing physiochemical properties and selective distribution of the carrier to absorb, embed, or interact with anthocyanins, which can effectively resolve issues with poor stability and limited uptake.

### Mechanisms underlying the anti-inflammatory effects of anthocyanins

At the cellular and molecular levels, anthocyanins and their metabolites have recently been shown to exert anti-inflammatory effects by activating the PI3K/Akt/eNOS, silent information regulator, and nuclear factor erythroid 2–related factor 2 pathways, while concurrently reducing the expression of toll-like receptor 4 and inhibiting the activation of the nuclear factor-κB (NF-κB) and p38 mitogen-activated protein kinase signaling pathways. Anthocyanins are further known to modulate antioxidant enzymes and impede the formation of reactive oxygen species (ROS) through the inhibition of the NAD(P)H oxidase 4 (NOX4) protein, which is the main catalytic component of NAD(P)H oxidase—an important source of ROS. This leads to decreased secretion of cytokines and chemokines by inflammatory cells through suppression of IκBα phosphorylation, which is known to further stimulate NF-κB activation and ROS production. These mechanisms, together with the ability of anthocyanins to activate Bim through the mitochondrial pathway (which promotes cell apoptosis), contribute to the growing body of evidence supporting their potential anticancer properties, particularly in the gastrointestinal tract because of their higher exposure and uptake in these tissues.

Further, anthocyanins also seem to suppress vascular smooth muscle cell proliferation and migration induced by platelet-derived growth factor or tumor necrosis factor-α (TNF-α) through inhibition of the focal adhesion kinase and extracellular signal-regulated kinase pathways. This contributes to the attenuation of vascular inflammation by reducing oxidized lipid formation, preventing leukocyte adhesion and transmigration and limiting macrophage uptake of deposited lipids via downregulation of CD36 and upregulation of ABCA1 and ABCG1. Similarly, inhibition of the c-Jun-N-terminal kinase and NF-κB pathways in the brain reduces microglia activation and induction of proinflammatory cytokine genes coding for TNF-α, IL-6, or MCP-1, in addition to cyclooxygenase-2, demonstrating the potential of anthocyanins to attenuate neuroinflammation.

## Author contributions

The authors responsibilities were as follows - TCW and MMG: design, writing, and final concept. All authors have read and approved the manuscript.

## Declaration of Generative AI and AI-assisted technologies in the writing process

The authors declare that no generative AI or AI-assisted technologies were used in the writing of this manuscript.

## Funding

The authors reported no funding received for this study.

## Conflict of interest

The authors report no conflicts of interest.
